# Langzeitverläufe der depressiven Erkrankung: Merkmale, Risikofaktoren und die definitorische Herausforderung des Therapieansprechens

**DOI:** 10.1007/s00115-024-01756-9

**Published:** 2024-10-14

**Authors:** Rebecca Paetow, Thomas Frodl

**Affiliations:** Klinik für Psychiatrie, Psychotherapie und Psychosomatik, Universitätsklinik Aachen, Pauwelsstr. 30, 52074 Aachen, Deutschland

**Keywords:** Depressive Störung, Therapieresistente Depression, Risikoerfassung, Biomarker, Zentrale Bildgebung, Depressive disorder, Treatment-resistant depression, Risk assessment, Biomarkers, Neuroimaging

## Abstract

**Hintergrund:**

Die Definition von Langzeitverläufen der Depression ist heterogen. Vor allem chronische und therapieresistente Verläufe bedeuten einen hohen Kostenfaktor und reduzieren Lebensqualität stark. Ausgehend von der pharmakotherapeutischen „therapieresistenten Depression“ (TRD) rücken immer mehr systemische Ansätze in den Vordergrund.

**Ziel der Arbeit:**

Dieses narrative Review bietet eine Übersicht zu Langzeitverläufen depressiver Erkrankungen, inklusive verschiedener Definitionen und Einflussfaktoren. Zudem wird eine Übersicht zur Biomarkerforschung des Therapieansprechens mit Fokus auf zentrale Bildgebung vorgestellt.

**Material und Methoden:**

Es erfolgte eine selektive Literaturrecherche in PubMed und Google Scholar für ein narratives Review. Besonders berücksichtigt wurden größere Kohortenstudien, systematische Reviews, Metaanalysen und Studien zur Prädiktion von Therapieansprechen.

**Ergebnisse:**

Chronische und therapieresistente Verläufe bedeuten eine relevante Reduktion von Lebensqualität sowie erhöhte gesundheitliche Risiken. Die Erfassung des Therapieansprechens ist eine definitorische Herausforderung: Eine Alternative zur TRD bietet die systemisch orientierte „schwer zu behandelnde Depression“ („difficult-to-treat depression“, DTD). Der Fokus bewegt sich damit fort von einer Symptomreduktion hin zu einer Kontrolle des Funktionsniveaus. Biomarkerforschung für Therapieansprechen bietet Potenzial, dient derzeit aber hauptsächlich dem theoretischen Erkenntnisgewinn.

**Diskussion:**

Die Erfassung von Langzeitverläufen depressiver Erkrankungen ist wichtig, aber auch komplex. Klinische Interventionen sollten daher ein kontinuierliches Monitoring miteinschließen und den Fokus auf den Erhalt von Lebensqualität legen.

**Zusatzmaterial online:**

Die Online-Version dieses Beitrags (10.1007/s00115-024-01756-9) enthält weitere Tabellen.

## Abstract (en)

**Background:**

The definition of long-term courses of depression is heterogeneous. Chronic and treatment-resistant courses, in particular, represent a high-cost factor and greatly reduce the quality of life. Based on the pharmacotherapeutic treatment-resistant depression (TRD), more and more systemic approaches are becoming important.

**Objective:**

This narrative review provides an overview of the long-term course of depressive disorders, including various definitions and influencing factors. In addition, an overview of biomarker research on treatment response with a focus on neuroimaging is presented.

**Material and methods:**

A selective literature search was conducted in PubMed and Google Scholar for a narrative review. Particular attention was given to larger cohort studies, systematic reviews, meta-analyses and studies on the prediction of treatment response.

**Results:**

Chronic and treatment-resistant courses mean a relevant reduction in the quality of life and increased health risks. The assessment of treatment response is a definitional challenge: An alternative to TRD is the systemically oriented difficult to treat depression (DTD). The focus is thus moving away from symptom reduction towards controlling the level of functioning. Biomarker research for treatment response offers potential but currently mainly serves to gain theoretical knowledge.

**Conclusion:**

Recording the long-term course of depressive illnesses is important, but also complex. Clinical interventions should therefore include a continuous monitoring and the focus on maintaining the quality of life.

Depressionen sind häufige Erkrankungen mit unterschiedlichen Verläufen. Unter den Langzeitverläufen sind chronische und therapieresistente Formen voneinander abzugrenzen. Eine definitorische Herausforderung bietet hier die Erfassung des Therapieansprechens: Als Alternative zur „therapieresistenten Depression“ (TRD) ist der Begriff „schwer zu behandelnde Depression“ (DTD) in den Vordergrund gerückt. Er geht über eine pharmakotherapeutische Definition hinaus und arbeitet mit einem multidimensionalen Ansatz. Die Biomarkerforschung zeigt vielversprechende Ergebnisse für die Prädiktion von Therapieansprechen.

Eine wichtige Längsschnittstudie aus Deutschland zu Prävalenz und Verlauf affektiver Störungen bietet die sog. „Studie zur Gesundheit Erwachsener in Deutschland“ (DEGS1). Sie ermöglicht eine bundesweite Einschätzung von Morbidität, Einschränkungsprofilen und Inanspruchnahme des Gesundheitssystems durch Erwachsene. Ihr liegt eine große Strichprobe mit 5317 Teilnehmern aus einer Altersspanne von 18 bis 79 Jahren zugrunde, die mit umfassenden klinischen Interviews beurteilt wurden. Laut dieser Längsschnittstudie [16] beträgt die 12-Monats-Prävalenz für unipolare Depressionen etwa 8,2 %, was wiederum einem Anteil von 5,3 Mio. Betroffenen in Deutschland entspricht, die in einem Zeitraum von 12 Monaten erkrankt sind. Auch wenn Depressionen in der Regel episodisch verlaufen, so ist davon auszugehen, dass etwa ein Drittel als chronisch zu bewerten ist [23].

In einer deutschen multizentrischen Langzeitstudie mit 782 Patient*innen zum 3‑Jahres-Verlauf der unipolaren Depression zeigte sich, dass 12 % der Patient*innen keine Remission erzielten. 52 % hatten einen fluktuierenden Verlauf und wechselten zwischen dem Status der Remission und Nichtremission [26]. Damit bestätigte diese Studie die Ergebnisse einer älteren Studie von 1998, der zufolge der Verlauf der unipolaren Depression sehr dynamisch und wechselhaft ist und sich verschiedene Schweregrade intraindividuell über den Langzeitverlauf abwechseln [18]. Eine 2020 publizierte Langzeitstudie mit 914 Teilnehmenden aus dem Kollektiv der „Netherlands Study of Depression and Anxiety“ (NESDA; [15]) zeigte, dass der Schweregrad – trotz Remission – fortbestehender Funktionsbeeinträchtigung mit der residualen depressiven Symptomatik korrelierte und dabei mit höherem Alter, stärkerem Vermeidungsverhalten, stärkerer Einschränkung bei Studienstart und späterer Remission assoziiert war.

Ziel dieses narrativen Reviews ist es zum einem, eine Übersicht zu Langzeitverläufen, inklusive definitorischen Herausforderungen, der depressiven Erkrankung zu geben sowie deren Einflussvariablen aufzuzeigen. Zum anderen soll dargestellt werden, welche Biomarker geeignet sein könnten, die Verläufe vorherzusehen, hier mit einem Fokus auf zentrale morphologische und funktionelle Bildgebung. Abschließend werden Schlussfolgerungen für das klinische Vorgehen präsentiert.

## Methodik

Es erfolgte eine selektive Literraturrecherche in PubMed und Google Scholar mit der Sucheingabe: (″Depressive Disorder, Major″ OR ″Major Depressive Disorder″ OR ″Depression″ OR ″Depressive Disorder″ OR ″Depression″) OR (″Chronic Depression″ OR ″Long-Term Depression″ OR ″Persistent Depressive Disorder″ OR ″Dysthymia″) OR (″Treatment-Resistant Depression″ OR ″Treatment-Resistant″ OR ″Therapy-Resistant″ OR ″Difficult-to-Treat Depression″ OR ″TRD″ OR ″DTD″) OR (″Risk Assessment″ OR ″Risk Factors″ OR ″Risk Assessment″ OR ″Risk Factors″ OR ″Predictors″) OR (″Biomarkers″ OR ″Genetic Markers″ OR ″Metabolic Markers″) OR (″Neuroimaging″ OR ″Magnetic Resonance Imaging″ OR ″MRI″ OR ″fMRI″ OR ″Brain Imaging″).

Besonders berücksichtigt wurden größere Kohortenstudien, systematische Reviews, Metaanalysen und Studien zur Prädiktion von Therapieansprechen. Zudem wurde die Nationalen Versorgungsleitlinie (NVL) miteinbezogen.

## Chronische Verlaufsform

**Abb. 1:**
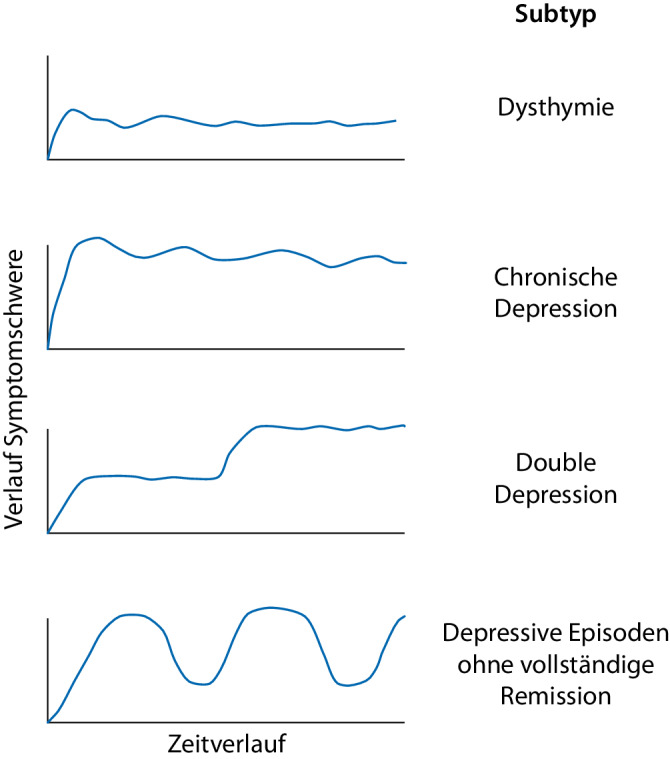
Formen chronischer Depression nach Zeitverlauf (x-Achse) und zunehmender Symptomschwere (y-Achse). (Mod. nach [6])

Die Definitionen der chronischen Depression unterscheiden sich bezüglich der Dauer von mindestens ein bis drei Jahren sowie bezüglich Verlauf und Schwere ([7], Abb. 1). Aufgrund der nicht unumstrittenen Gültigkeit dieser Einteilung erfolgt im DSM‑5 die gänzliche Zusammenfassung aller chronischen Verlaufsformen in „persistierende depressive Störung“. Im ICD-10 wird im Kapitel F33 bisher nicht zwischen chronisch und rezidivierend differenziert. Im ICD-11 hingegen erfolgt eine getrennte Verschlüsselung (6A71, 6A80). Weiterhin wird im ICD-11 eine unvollständig remittierte und länger als zwei Jahre anhaltende depressive Episode als „partiell remittiert“ und nicht mehr als „persistierend“ kodiert.

Eine Art Vorstufe bezüglich klinischer Schwere für die Depression bietet die sog. Dysthymie. Nach ICD-10 (F34.1) handelt es sich hier um eine chronische, über mehrere Jahre bestehende depressive Verstimmung, die auch phasisch bzw. in Episoden verlaufen kann. Jedoch muss für die Dysthymie beachtet werden, dass sie in ihren Episoden nicht stark genug ist, die Kriterien einer leichten, mittelgradigen oder schweren depressiven Episode zu erfüllen. Eine Dysthymie wird auch im ICD-11 weiterhin dann kodiert, wenn die depressiven Symptome nicht schwer genug für die Kriterien einer leichten, mittelgradigen oder schweren Episode sind (6A72).

Eine sog. „double depression“ liegt vor, wenn eine Dysthymie von einer akuten depressiven Episode überlagert wird.

## Therapieresistente Verlaufsform

Definitorisch abzugrenzen unter den Langzeitverläufen sind die chronische Depression und die therapieresistente Depression, im Englischen „treatment-resistant depression“ (TRD), für deren definitorische Anwendung bis dato kein eindeutiger Konsens vorliegt, da sie nach Therapieansprechen auf Antidepressiva festgelegt ist: Mit dem Begriff der TRD wird üblicherweise auf eine pharmakologische Definition verwiesen, die von der amerikanischen Food and Drug Administration (FDA) und der European Medicines Agency (EMA) 2018 implementiert wurde. Nach jener häufig verwendeten Definition liegt eine therapieresistente Depression dann vor, wenn Patient*innen bei mindestens zwei Antidepressiva aus verschiedenen Wirkstoffklassen bei ausreichender Einnahmedauer sowie Dosierung und Therapietreue keine Response gezeigt haben [14]. Laut einer retrospektiven Beobachtungsstudie aus Ungarn liegt bei 8,3 % aller unipolaren Depressionen eine therapieresistente Depression vor: Es wurde hier eine Datenbank aus dem Jahre 2009 bis 2015 mit 99.531 Depressionspatient*innen ausgewertet, von denen 8268 die Kriterien für eine TRD erfüllten [5].

Die Autor*innen um Cipriani führten 2018 ein systemisches Review und eine Metaanalyse zu 522 Studien, in denen Depressionspatient*innen ab 18 Jahren in placebokontrollierten Studien untersucht wurden, durch. Es wurden hier 21 antidepressive Substanzen erfasst. Nach der metaanalytischen Auswertung der sog. Odds Ratios lag die Rate der nichtausreichenden Response über alle pharmakologischen Substanzen gemittelt bei 43 % [4].

Die Bereiche chronisch und therapieresistent lassen sich allgemein nicht klar abgrenzen, da sie sowohl einzeln als auch gleichzeitig bei Patient*innen vorliegen können. Aus einer rein definitorischen Perspektive wäre z. B. eine seit drei Jahren anhaltende schwere depressive Episode ohne bisherige Leitlinienpsychopharmakotherapie, weil der Patient bei Unverträglichkeit eine adäquate Einstellung ablehnt, zwar chronisch, aber nicht therapieresistent. Der Begriff der (Nicht‑)Therapieresistenz scheint in diesem Falle nicht zielführend und die Situation des Beispiel-Patienten nicht angemessen zu repräsentieren. Allgemein sollten daher die Behandlungsziele für chronische und therapieresistente Depressionen an den Endpunkten der Funktionsfähigkeit sowie Lebensqualität der Patient*innen ansetzen: Keitner und Mansfield [20] empfahlen bereits 2012, die TRD als eine chronische Erkrankung, die zwar nicht im Sinne einer Restitution zum Ausgangsniveau gänzlich geheilt werden kann, durch eine systemische Erfassung inklusive Therapieplanung aber modulierbar ist, zu verstehen. Daher empfehlen die Autor*innen auch ein Umdenken: Therapie soll nicht alleine darauf abzielen die klinischen Symptome der Betroffenen zu mildern, sondern sie mit ihrem gegebenen Funktionsniveau haltbar in alltägliche Strukturen zu integrieren [20].

Daher sollte nach aktuellem Diskurs in der Literatur Therapieansprechen multifaktoriell und nicht allein unter pharmakotherapeutischen Gesichtspunkten erfasst werden. Beachtet werden muss auch das potenzielle Studienbias, dass der Anteil an Patient*innen mit therapieresistenter Depression bei einer anzunehmenden Vorselektion in spezialisierten klinischen Zentren größer ist, als in Studien, die auf administrativen Daten basieren. Schließlich umfassen jene administrativen Daten auch mehr ambulante Kollektive, die weniger stationäre und spezialisierte Versorgungssystem in Anspruch nehmen. Kritisch an der Definition der TRD ist zudem die Implikation, dass der Wechsel innerhalb einer Antidepressivaklasse weniger effektiv ist als zwischen zwei Klassen, was sich bisher nicht bewahrheiten ließ: In einer Metaanalyse aus dem Jahre 2018 zeigten sich in einer Auswertung von vier (enger gefasste Auswertung) bzw. acht (weiter gefasste Auswertung) randomisierten und verblindeten Studien keine Überlegenheit eines Wechsels im Vergleich zur Fortführung eines Antidepressivums [2].

Die Autor*innen um McIntyre fassen in ihrer Übersichtsarbeit aus dem Jahre 2023 [21] weitere Interventionen zum Management der TRD zusammen: Hiernach bietet im therapeutischen Kontext Esketamin intranasal und intravenös eine wirksame Behandlung von TRD. Manche Antipsychotika der zweiten Generation, wie z. B. Aripiprazol, Quetiapin und Olanzapin, zeigen einen wirksamen additiven Effekt als augmentierende Psychopharmaka. Auch Neurostimulationsverfahren, wie repetitive transkranielle Magnetstimulation (rTMS) und Elektrokonvulsionstherapie (EKT), bieten ein effektives Therapiemanagement für die TRD. Die Evidenzlage für eine Kombination von Antidepressiva und den Wechsel der Antidepressivaklasse bei TRD ist gemischt [21]. Psychotherapie in Kombination mit Pharmakotherapie zeigt sich als wirksam zur Linderung depressiver Symptome bei einer TRD. Digitale Anwendung werden derzeit noch erforscht und bieten potenziell eine wertvolle und zukünftige Therapieergänzung. In der NVL werden sie bereits empfohlen [13].

## Einflussfaktoren und Merkmale chronischer und therapieresistenter Verläufe

Chronische und therapieresistente Verlaufsformen unterschieden sich nach Studienlage in mehreren Merkmalen bezüglich möglicher Einflüsse und Folgen. Zugleich reduzieren beide Verlaufsformen die Lebensqualität der Betroffenen erheblich. Daher soll hier auf jene verschiedenen Merkmale eingegangen werden:

Die Vorhersagbarkeit für ein dauerhaftes Nichtansprechen auf eine Therapie (Tabelle e1 im Onlinezusatzmaterial) oder für eine Chronifizierung (Tabelle e2 im Onlinezusatzmaterial) ist vergleichbar mit den Risiken für die Entstehung einer Depression und mit den Risiken für Rückfälle und Rezidive [13]:

Im Vergleich zu einem episodischen Verlauf sind Patient*innen mit chronischen Verläufen häufiger von komorbiden psychischen Erkrankungen betroffen, darunter vor allem Angststörungen, Alkoholabhängigkeit und Persönlichkeitsstörungen laut einer nationalen Querschnittsbefragung aus Australien mit Daten von 8841 Haushalten aus dem Jahre 2007 [23]. In einem systematischen Review von 2010 mit Einschluss von 5192 Teilnehmenden aus 25 Primärstudien zeigte sich bei chronischen Verläufen eine stärkere Einschränkung im Funktionsniveau, vor allem durch mangelnde soziale Integration [12]. Diese Faktoren erschweren einen geregelten Behandlungsablauf und begünstigen damit Therapieresistenz. Hinzu kommt bei den chronischen Depressionen, dass sich allgemein eine depressive Erkrankung häufig früher bei den Betroffenen manifestiert und die Chronifizierung zu einer höheren Beanspruchung des Gesundheitssystems führt, inklusive stationärer Behandlungen [23]. In einer multizentrischen Studie mit 604 Patient*innen mit Depression, erfuhren 60–70 % aller chronisch Betroffenen (*n* = 65) früh ein interpersonelles Trauma bzw. negative Kindheitserfahrungen, wie z. B. emotionale und körperliche Vernachlässigung [28].

An einer europäischen Gesundheitsbefragung von 2019 mit 52.060 Personen hatten 3308 (6,4 %) in den letzten 12 Monaten eine Depression und wiederum 18,8 % litten unter einer therapieresistenten Form. Patient*innen mit therapieresistenter Depression litten signifikant häufiger als jene mit nichttherapieresistenter Depression unter Anämie, chronischer Herzinsuffizienz und rheumatoider Arthritis. Therapieresistente Patient*innen berichten zudem häufiger über eine positive Familienanamnese für Depression, Angst sowie Suizidabsichten. Sie hatten weiterhin signifikant höhere Fehlzeiten und stärkere Beeinträchtigungen an ihrem Arbeitsplatz als die nichttherapieresistenten Patient*innen [17]. In einer weiteren retrospektiven Auswertung zuvor zitierter Datenbank von 2009 bis 2015 aus Ungarn hatten Patient*innen mit therapieresistenter Depression eine signifikant höhere Wahrscheinlichkeit für neurotische, stressbedingte und somatoforme Störungen, Autoimmunerkrankungen, kardio- oder zerebrovaskuläre Erkrankungen, Schilddrüsenerkrankungen und selbstverletzendes Verhalten als jene ohne therapieresistente Verlaufsform [5].

Für weitere Erkenntnisse im europäischen Raum wurde 2021 eine Kohortenstudie mit Daten von 411 Patient*innen aus der klinischen Routine durchgeführt. In dieser Kohortenstudie wurden die Behandlungsergebnisse von Patient*innen mit mittelschwerer bis schwerer therapieresistenter Depression, die eine neue Therapie begannen, über einen Zeitraum von bis zu 21 Monaten verfolgt: Im Durchschnitt waren die Patient*innen bereits für 2,6 Jahre lang in ihrer gegenwärtigen depressiven Episode. Zwei Drittel dieser Patient*innen hatten eine mittelschwere depressive Episode, ein Drittel eine schwere depressive Episode. 19,3 % der Patient*innen hatten zudem bei Einschluss eine laufende Psychotherapie. 61,6 % der Patienten gaben schwere bis extreme Beeinträchtigungen am Arbeitsplatz durch die gegenwärtige Depression an [11].

Ein weiterer Aspekt bei therapieresistenter Depression ist die Komorbidität mit Persönlichkeitsstörungen: In einer Psychotherapieverlaufsstudie hatten bei Einschluss ca. 37 % der Patient*innen eine Cluster-C-, 16 % eine Cluster-B- und 8 % eine Cluster-A-Persönlichkeitsstörung nach DSM‑5. Vierzehn Monate nach der Beendigung der Therapie zeigten 63,79 % der Patient*innen keine Persönlichkeitsstörung mehr [27]. Diese geringe Stabilität einer Diagnose der Persönlichkeitsstörung allgemein im Verlauf einer depressiven Erkrankung ist zudem belegt in einer Therapiestudie von 2010 mit 149 Patient*innen [22].

Chronische und therapieresistente Depressionen sind in ihren Risikoprofilen und Prädiktoren scheinbar verschieden: Chronische Depressionen sind häufig mit frühkindlichen Traumata und negativen Kindheitserfahrungen assoziiert, was auf widrige Lebensumstände im Kindes- und Jugendalter und einer resultierenden dysfunktionalen Entwicklung hinweisen könnte. Hingegen sind bei therapieresistenten Depressionen körperliche Erkrankungen stärker ausgeprägt. Möglich wäre daher, dass für therapieresistente Depressionen genetische Dispositionen mitverantwortlich sind. Diese hypothetische Unterscheidung gilt es, anhand von Metaanalysen und prospektiven Studien zu überprüfen. Ein potenzieller Bias könnte durch unterschiedliche Studiendesigns hinsichtlich der untersuchten Variablen bei chronischer und therapieresistenter Depression bedingt sein.

## Schwer zu behandelnde Verlaufsform

**Abb. 2:**
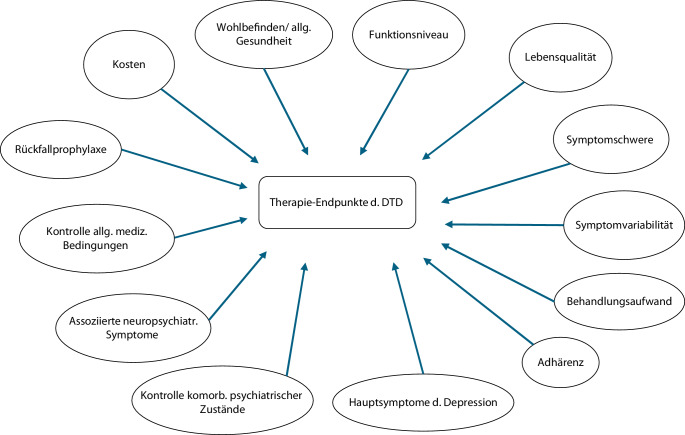
Wichtige klinische Endpunkte für die Therapieforschung der „difficult-to-treat depression“ (DTD). (Nach [25])

Eine moderne Alternative zur Erfassung und Definition von Verlaufsformen, die nicht hinreichend auf Therapie ansprechen, bildet die schwer zu behandelnde Depression, im Englischen „difficult to treat depression“ (DTD), nach Rush [25]. Diese geht über das pharmakologische Therapieansprechen hinaus und beachtet damit auch Patient*innen, die primär mit Psychotherapie behandelt wurden und nicht adäquat darauf ansprachen. Im Kontrast zur TRD berücksichtigt die DTD für das Therapieansprechen auch die alltägliche Einschränkung und Lebensqualität der Patient*innen. Damit definiert sie eine relevante Untergruppe an Betroffenen, die entweder bei chronifizierten Beschwerden keine relevante Linderung unter Therapie finden, oder, trotz einer Besserung ihrer Symptomlast, keine hinreichende Besserung in ihrer Lebensqualität und Alltagsstruktur erhalten [25]. Des Weiteren fordert die Definition einer DTD eine multifaktorielle Erfassung der Krankheitslast mit psychiatrischen, allgemeinmedizinischen und neuropsychologischen Verlaufsbeobachtungen. Schließlich sind Patient*innen mit einer DTD systemisch erkrankt. Aus der Perspektive einer DTD werden damit unterschiedliche Aspekte des Krankheitsverlaufes berücksichtigt und bieten damit eine wertvolle Ergänzung für Diagnosemanuale und die TRD (Tab. 1, Abb. 2).

**Tab. 1:** Definitorische Übersicht zur therapieresistenten und schwer zu behandelnden Depression

Therapieresistente Depression („treatment resistent depression“ TRD; nach [14])	*Pharmakologisch*: Keine klinische Besserung unter Einnahme zweier Antidepressiva aus verschiedenen Wirkstoffklassen, – die je ausreichend lange, – ausreichend hoch dosiert und – ausreichend adhärent verwendet wurden
Schwer zu behandelnde Depression („difficult to treat depression“, DTD; nach [25])	*Heuristischer Sammelbegriff:* – Keine klinische Besserung bei einer chronischen Depression trotz leitliniengerechter Therapie oder – Trotz Therapieansprechen keine hinreichende systemische Besserung
	*Dies führt u.* *a. zu* – Reduktion von Lebensqualität – Erhöhter Beanspruchung des Gesundheitswesens – Erhöhter Mortalität und Morbidität – Einschränkung verschiedener Endpunkte (siehe auch Abb. 2)

Eine Übersichtsarbeit zu den Charakteristika der DTD bieten die Autor*innen um Rush aus dem Jahre 2022 [25]: Betroffene können, auch wenn eine leitliniengerechte Therapie durchgeführt wurde bzw. angenommen wird, keine Remission erfahren. Dabei liegen in der Regel chronisch die Symptome einer Depression vor, wobei diese durch Therapie nicht hinreichend gemildert werden oder aber trotz Therapieansprechen keinen hinreichenden Benefit für die Betroffenen bieten. Durch die DTD kommt es zu einer nachhaltig reduzierten Lebensqualität. Weiterhin wird sowohl ambulant als auch stationär vermehrt das Gesundheitswesen beansprucht, was wiederum mit hohen finanziellen und über Jahre anhaltenden Kosten einhergeht. Zusätzlich ist die DTD mit erhöhter Mortalität und Morbidität assoziiert und Betroffene weisen häufiger eine positive Familienanamnese für psychische Erkrankungen auf und sind unter anderem auch häufiger von aversiven Ereignissen bzw. Traumatisierung in der frühen Biographie betroffen. Schätzungsweise liegt bei 15–25 % aller Patient*innen mit einer Depression eine DTD vor.

Kernforderungen zur Therapie bestehen darin, bei Verdacht einer DTD zuerst die aktuelle depressive Episode unter medizinischen und neuropsychologischen Gesichtspunkten zu erfassen und leitliniengerecht zu behandeln. Wenn hierunter keine relevante Besserung für die Betroffenen eintritt, gilt die DTD als bestätigt und der therapeutische Fokus sollte sich fort von der symptomatischen Remission hin zu Erhaltung und Stärkung der psychosozialen Funktion verlagern.

Nach gegenwärtiger Sachlage ist der Forschungsbedarf für die DTD hoch, zum einen, weil sie einen hohen Leidensdruck bedeutet, zum anderen, weil Patient*innen mit einer DTD aufgrund erhöhter Risiken, z. B. für Suizidalität, eher aus Studienprotokollen ausgeschlossen werden [25]. Zudem ist eine Vereinheitlichung der Taxonomie und Definition von Subgruppen innerhalb der DTD für das Planen von Studien notwendig [25].

## Forschung zu Therapieprädiktion und Verlauf

Im Rahmen personalisierter Medizin, der Vorbeugung von Chronifizierung und Therapieresistenz sowie des Wunsches nach individualisierten Therapieformen bei Depression werden potenzielle Prädiktoren für das Therapieansprechen intensiv beforscht. Für das Ansprechen auf Antidepressiva wurden diverse biologische Prädiktoren untersucht, darunter Metabolite zentralnervöser Transmitter, Enzymaktivität im Transmitterstoffwechsel, neuroendokrinologische und neurophysiologische Verfahren, unter anderem mit Rapid-eye-movement(REM)-Schlaflatenz, elektrodermaler Aktivität und verschiedenen Elektroenzephalogramm(EEG)-Parametern sowie struktureller Magnetresonanztomographie (MRT) und funktioneller MRT (fMRT). Auf die letzteren beiden soll hier näher eingegangen werden, wobei die Studien nicht spezielle Kollektive mit therapieresistenten Patient*innen untersuchten:

Hirnstrukturelle Veränderungen im Hippokampus konnten per MRT in einer Studie mit 30 Versuchspersonen (initial stationäre Patient*innen mit der Diagnose einer Depression) und 30 Kontrollen anhand von Korrelationsanalysen mit einem schlechteren Therapieverlauf in Verbindung gebracht werden [7]. In einer prospektiven 3‑Jahres-Studie der gleichen Kohorte konnte morphologisch das Volumen des Hippokampus als potenzieller Prädiktor per multivariater Varianzanalyse (MANOVA) für Therapieansprechen identifiziert werden [8]. In einer weiteren bildmorphologischen Studie aus dem Jahre 2004 mit 31 Versuchspersonen (Patient*innen mit Diagnose einer Depression ohne Pharmakotherapie, davon 21 akut-depressiv und 10 remittiert) und 31 Kontrollen zeigte die Region des Hippokampus in einer Kovarianzanalyse (ANCOVA) sogar das Potenzial, zwischen dem Status einer aktuellen depressiven Episode und einer gegebenen Remission zu differenzieren [3].

Auch mit fMRT-Untersuchungen, die es erlauben, bestimmte emotionale und kognitive Prozesse gezielter zu untersuchen, wurde versucht, Prädiktoren für das Ansprechen auf Psychotherapie und Psychopharmakotherapie zu finden. Überaktivität in der Amygdala und Unteraktivität im anterioren zingulären Kortex waren mit Response auf kognitive Verhaltenstherapie in einer Studie mit 16 Versuchspersonen ohne Antidepressivum und 16 Kontrollen in einer Korrelationsanalyse assoziiert [9]. In einer fMRT-Studie, in die 80 Patient*innen mit depressiver Störung und 34 gesunde Proband*innen eingeschlossen wurden [31], wurden die Patient*innen, die ohne Antidepressivum waren oder vorher ihre Antidepressivaeinnahme ausgeschlichen hatten, in einen von drei pharmakotherapeutischen Behandlungsarmen (Escitalopram, Venlafaxin oder Sertralin) randomisiert. Die statistische Analyse möglicher prädiktiver Eigenschaften von Amygdalaaktivität erfolgte per allgemeinem linearem Modell (GLM). Responder auf Antidepressiva waren hier durch niedrigere Amgygdalaaktivität bei subliminalen positiven Stimuli gekennzeichnet. Amygdalaüberaktivität auf die Präsentation trauriger Gesichter war speziell prädiktiv für Nonresponse auf Venlafaxin. Die Genauigkeit der Prädiktion zwischen Respondern und Nonrespondern betrug 75 %.

Weitere repräsentative Verlaufsergebnisse bietet die NESDA-Studie [29], in der zwischen 2005 und 2007 233 Patient*innen mit Depressionen und Angsterkrankungen eingeschlossen und auch bildmorphologisch untersucht wurden. Die bildgebenden Untersuchungen beinhalteten Follow-up-Studien im Intervall von 2 und 9 Jahren. Hier konnten allgemeine morphologische und neurokognitive Abweichungen verglichen mit gesunden Kontrollen festgestellt werden. Die Daten zeigten vor allem bei der Verarbeitung von Emotionen ein hohes Potenzial für die Beurteilung von Langzeitverläufen affektiver Störungen. Zudem erlaubte die im fMRT gemessene Gesichtsverarbeitung depressiver Patient*innen der NESDA-Kohorte eine Aufteilung in Trajektorien, also statistische Verlaufsformen (chronisch-depressiv, graduell genesend, schnell remittierend; [29]).

In Deutschland ist die Marbug-Münster Affective Disorders Cohort Study (MACS) der FOR 2107 (DFG-Forschergruppe 2107) ein repräsentatives Verbundprojekt für die Erforschung affektiver Störung, inklusive funktioneller Bildgebung bei Depressionen [30]. 2023 [10] wurden hier über ein Verbundprojekt Machine-Learning-Analysen auf 3377 fMRT-Resting-State-Datensätze aus zwei multizentrischen Studien angewendet. Hyperkonnektivität im Thalamus zeigte sich als prominente Signatur für Depressionen. Die allgemeine Resting-State-Konnektivität konnte zu etwa 60 % Depressionen detektieren, was vermutlich durch die Heterogenität von Depressionen bedingt war.

Somit könnten fMRT und MRT eine Hilfe sein, bessere Vorhersagen über Verlauf samt Therapieansprechen zu treffen. Jedoch muss berücksichtigt werden, dass für die Validierung dieser Ergebnisse weitere und umfassende Kohortenstudien notwendig sind.

Abseits der (f)MRT-Forschung wird im Rahmen von Fortschritt und Verfügbarkeit genetischer Methoden derzeit vor allem die Pharmakogenetik beforscht: Hier konnte unter anderem in einer Metaanalyse mit gepoolten Analysen zu Response- und Remissionsraten über 49 Studien [24] das L‑Allel des Polymorphismus der Promotorregion des Serotonintransporter-Gens (5-HTTLPR) als potenzieller Prädiktor für Therapieansprechen und Remission identifiziert werden, wobei aber Effekte der ethnischen Herkunft und des biologischen Geschlechts berücksichtigt werden müssen. In einer anderen Studie mit 225 Patient*innen [19] zeigte sich unter Nutzung von Machine Learning und Clusteranalysen, dass komplexe Interaktionsanalysen mit genetischen und klinischen Variablen die Vorhersagekraft für Therapieansprechen erhöhen können. Hier zeigte sich ein Modell mit 3 Single-Nukleotid-Polymorphismen (SNPs) und einer klinischen Variable am vielversprechendsten.

Allgemein ist der Nutzen neurobiologischer Forschungsergebnisse derzeit auf den theoretischen Erkenntnisgewinn begrenzt. Vor einer Anwendung in der klinischen Routine müssten potenzielle Biomarker auch in großen und repräsentativen klinischen Studien untersucht werden sowie auch neue Pharmaka in Zulassungsstudien. Diese sind entsprechend zeitaufwendig. Ein valider und ökonomischer Transfer in die diagnostische Routine ist deshalb bisher nicht absehbar, aber erstrebenswert, um die Therapie gleich zu Beginn nach Diagnosestellung anhand eines individuellen Responseprofils und nicht erst nach langjährigem Verlauf zu planen. Damit kann zukünftig das Risiko einer Chronifizierung weiter minimiert werden.

## Planung von Diagnostik und Therapie in der Praxis

Im Folgenden soll in Anlehnung an die NVL [13] ein Algorithmus bei Erstdiagnose, alternativ Erstkontakt bei gegebener Diagnose einer Depression, zu Diagnostik und Therapieplanung vorgestellt werden. Ziel ist es, durch kontinuierliches Monitoring und eine vorausschauende Therapieplanung den Übergang in einen Langzeitverlauf, allen voran in eine DTD, rechtzeitig zu erkennen. Somit kann bei festgestellter DTD das Therapieschema entsprechend angepasst werden.

1. Beim Verdacht einer Depression steht zu Beginn die standardisierte Diagnostik sowie die Erfassung von Risikofaktoren für Chronifizierung und ungünstiges Ansprechen/Nichtansprechen der Therapie.
2. Bei begründeter Diagnosestellung einer Depression folgt neben der Psychoedukation^1^ ein klares Behandlungskonzept unter Berücksichtigung der Leitlinie [13] und der Bedürfnisse der Patient*innen im Rahmen partizipativer Entscheidungsfindung mit folgenden Punkten [1]:
  Welche Therapieverfahren inklusive Medikation?
  Welche Reihenfolge der Therapieverfahren?
  Welche Dauer für welches Verfahren?
  Zu welchen festen Zeitpunkten erfolgt die systemische Überprüfung des Therapieerfolges? (Siehe Tabelle e3 im Onlinezusatzmaterial.)

## Fazit für die Praxis

- Depressionen sind häufige, heterogene Erkrankungen und werden bezüglich ihrer Folgen unterschätzt. Schätzungsweise ein Drittel verläuft chronisch. Die Erfassung der Langzeitverläufe bietet definitorische und methodische Herausforderungen.
- Von Langzeitverläufen Betroffene haben ein relevantes Risiko für diverse Einschränkungen und weitere psychiatrische und körperliche Erkrankungen.
- Eine Routinedefinition für Langzeitverläufe bietet die therapieresistente Depression (TRD), die pharmakotherapeutisch ausgerichtet ist.
- Eine multidimensionale Alternative zur TRD bietet die DTD („difficult-to-treat depression“), mit der sich das Therapieziel weg von einer Symptomreduktion hin zu einer Symptomkontrolle bewegt.
- Behandelnde sollten Betroffene diagnostisch multidimensional erfassen und für die Therapie klare Verlaufsbeurteilungen integrieren.
- Erkenntnisse aus der Biomarkerforschung, vor allem der f(funktionellen)MRT-Forschung, zeigen Potenzial für die Verlaufserfassung, dienen derzeit aber noch vor allem dem theoretischen Erkenntnisgewinn.

## Supplementary Information

Tabelle e1: Prädiktoren für ungünstiges Ansprechen/Nichtansprechen bei depressiven Störungen; Tabelle e2: Prädiktoren eines chronischen Verlaufs depressiver Störungen; Tabelle e3: Auswahl psychometrischer Tests zur Diagnostik und Wirkungsprüfung depressiver Störungen

## Abkürzungen

ANCOVA: „Analysis of covariance“, Kovarianzanalyse
BAG Selbsthilfe e. V.: Bundesarbeitsgemeinschaft Selbsthilfe von Menschen mit Behinderung, chronischer Erkrankung und ihren Angehörigen
BAPK e. V.: Bundesverband der Angehörigen psychisch Kranker
DEGS1: Studie zur Gesundheit Erwachsener in Deutschland, erste Erhebungswelle
DSM‑5: Diagnostic and Statistical Manual of Mental Disorders, Diagnostisches und Statistisches Manual Psychischer Störungen, 5. Auflage
DTD: „Difficult-to-treat depression“, schwer zu behandelnde Depression
EEG: Elektroenzephalogramm
EKT: Elektrokonvulsionstherapie
EMA: European Medicines Agency, Europäische Arzneimittelagentur
FDA: U.S. Food and Drug Administration, US-Behörde für Lebens- und Arzneimittel
fMRT: Funktionelle Magnetresonanztomographie
FOR 2107: DFG-Forschergruppe 2107
GLM: „General linear model“, allgemeines lineares Modell
5‑HTTLPR: „5-Hydroxytryptamin-transporter linked polymorphic region“, Serotonin-Transporter-Promoter-Polymorphismus
ICD-10: International Classification of Diseases, Internationale statistische Klassifikation der Krankheiten und verwandter Gesundheitsprobleme, 10. Revision
ICD-11: International Classification of Diseases, Internationale statistische Klassifikation der Krankheiten und verwandter Gesundheitsprobleme, 11. Revision
MACS: Marburg-Münster Affective Disorders Cohort Study
MANOVA: „Multivariate analysis of variance“, multivariate Varianzanalyse
MRT: Magnetresonanztomographie
NESDA: Netherlands Study of Depression and Anxiety, Studie der Niederlande zu Depressionen und Angsterkrankungen
NVL: Nationale Versorgungsleitlinie Unipolare Depression
REM: „Rapid eye movement“
rTMS: Repetitive transkranielle Magnetstimulation
SNP: „Single-nucleotide polymorphism“, Einzelnukleotidpolymorphismus
SNRI: „Serotonin noradrenalin reuptake inhibitor“, Serotonin-Noradrenalin-Wiederaufnahmehemmer
TRD: „Treatment-resistent depression“, Therapieresistente Depression

## Footnotes

1 Anmerkung: Für die Psychoedukation sei auch auf die Patientenblätter der NVL [13] verwiesen. Betroffene und Angehörige finden hier auch örtliche und überregionale Selbsthilfeorganisationen, wie die Deutsche Depressionsliga, der BAPK e. V. und die BAG Selbsthilfe e. V.
