# Supplementary material for: Long-term courses of major depressive disorder: Characteristics, risk factors and the definitional challenge of treatment response
Source: Nervenarzt. 2024 Oct 14;96(1):37–45. [Article in German] doi: 10.1007/s00115-024-01756-9 (PMC11772401; doi:10.1007/s00115-024-01756-9)
Supplement: Supplementary file 1 — Tabelle e1: Prädiktoren für ungünstiges Ansprechen/Nichtansprechen bei depressiven Störungen; Tabelle e2: Prädiktoren eines chronischen Verlaufs depressiver Störungen; Tabelle e3: Auswahl psychometrischer Tests zur Diagnostik und Wirkungsprüfung depressiver Störungen [file 115_2024_1756_MOESM1_ESM.pdf]

Langzeitverläufe der depressiven Erkrankung

Merkmale, Risikofaktoren und die definitorische Herausforderung des Therapieansprechens

Rebecca Paetow, Thomas Frodl

Klinik für Psychiatrie, Psychotherapie und Psychosomatik, Universitätsklinik Aachen, RWTH Universität Aachen, Aachen

**Tabelle e1: Prädiktoren für ungünstiges Ansprechen/ Nichtansprechen bei depressiven Störungen**
Genetische Faktoren
Klinische Faktoren<br>- Niedriges Erkrankungsalter<br>- Längere Dauer der depressiven Episode<br>- Höhere Anzahl an wiederkehrenden Episoden<br>- Melancholische Merkmale<br>- Anzahl der Hospitalisierungen<br>- Erhöhtes Suizidrisiko
Psychische Komorbidität, darunter<br>- Angststörungen<br>- Substanzbezogene Störungen<br>- Persönlichkeitsstörungen
Behandlungsbedingte Faktoren<br>- Ausbleibendes frühes Ansprechen<br>- Höhere Anzahl der Therapieversuche mit Antidepressiva<br>- Höhere Antidepressiva-Dosis
Soziodemographische Faktoren<br>- Familienstand
Anmerkung: nach NVL [13]; die Liste stellt eine Auswahl der in systematischen Übersichtsarbeiten identifizierten Prädiktoren dar, wobei für die einzelnen Faktoren nur sehr geringe und teilweise widersprüchliche Evidenz vorliegt

**Tabelle e2: Prädiktoren eines chronischen Verlaufs depressiver Störungen**
Höhere familiäre Raten an Depressionen, insbesondere chronische Depressionen
Früherer Krankheitsbeginn
Negative Kindheitserfahrungen<br>- Misshandlung<br>- Missbrauch<br>- Vernachlässigung
Belastende Lebensumstände<br>- Stress<br>- Armut<br>- Arbeitslosigkeit
Neurotizismus
Geringer Selbstwert
Grübeln
Kognitive Reaktivität
Anmerkung: nach NVL [13]

**Tabelle e3 nach [13]: Auswahl psychometrischer Tests zur Diagnostik und Wirkungsprüfung depressiver Störungen**	
Selbstbeurteilung	Fremdbeurteilung
Patient Health Questionnaire-Depression (PHQ-9)	Hamilton-Depression-Rating-Skala (HDRS)
Beck-Depressionsinventar (BDI-II)	Bech-Rafaelsen-Melancholie-Skala (BRMS)
Hospital Anxiety and Depression Scale (HADS)	Montgomery-Asberg Depression Rating Scale (MADRS)
Geriatrische Depressionsskala (GDS)	Inventar depressiver Symptome (IDS-C), Quick Inventory of Depressive Symptomatology – clinician-rated (QIDS-C)
Fragebogen zur Depressionsdiagnostik (FDD-DSM-IV)	
Allgemeine Depressionsskala (CES-D)	
Anmerkung: Die Auswahl der aufgeführten Tests ist nach [13] konsensbasiert. Details, Schwellenwerte und weitere Literatur siehe dort. Für die Testverfahren können Lizenzgebühren anfallen. PHQ-9 und GDS sind lizenzfrei	
